# Cortical Thinning of Motor and Non-Motor Brain Regions Enables Diagnosis of Amyotrophic Lateral Sclerosis and Supports Distinction between Upper- and Lower-Motoneuron Phenotypes

**DOI:** 10.3390/biomedicines9091195

**Published:** 2021-09-10

**Authors:** Stefano Ferrea, Frederick Junker, Mira Korth, Kai Gruhn, Torsten Grehl, Tobias Schmidt-Wilcke

**Affiliations:** 1 Institute of Clinical Neuroscience and Medical Psychology, Medical Faculty, Heinrich-Heine-University Dusseldorf, 40225 Dusseldorf, Germany; Frederick.Junker@ruhr-uni-bochum.de (F.J.); tobias-schmidt-wilcke@t-online.de (T.S.-W.); 2 Evangelisches Krankenhaus Hattingen, 45525 Hattingen, Germany; Mira.Gajsar@gmx.de; 3 Neuro Center Mettmann, 40822 Mettmann, Germany; k.gruhn@n-c-m.de; 4 ALS Outpatient Clinic, Alfried Krupp Krankenhaus Rüttenscheid, 45131 Essen, Germany; torsten.grehl@krupp-krankenhaus.de; 5 Neurologisches Zentrum, Bezirksklinikum Mainkofen, 94469 Deggendorf, Germany

**Keywords:** cortical thinning, multivariate analysis, amyotrophic lateral sclerosis, upper motoneuron, lower motoneuron

## Abstract

Background: Amyotrophic lateral sclerosis (ALS) is a neurodegenerative disorder clinically characterized by muscle atrophy and progressive paralysis. In addition to the classical ALS affecting both the upper and lower motoneurons (UMN and LMN), other subtypes with the predominant (or even exclusive) affection of the UMN or LMN have been identified. This work sought to detect specific patterns of cortical brain atrophy in the UMN and LMN phenotypes to distinguish these two forms from the healthy state. Methods: Using high-resolution structural MRI and cortical thickness analysis, 38 patients with a diagnosis of ALS and predominance of either the UMN (*n* = 20) or the LMN (*n* = 18) phenotype were investigated. Results: Significant cortical thinning in the temporal lobe was found in both the ALS groups. Additionally, UMN patients displayed a significant thinning of the cortical thickness in the pre- and postcentral gyrus, as well as the paracentral lobule. By applying multivariate analyses based on the cortical thicknesses of 34 brain regions, ALS patients with either a predominant UMN or LMN phenotype were distinguished from healthy controls with an accuracy of 94% and UMN from LMN patients with an accuracy of 75%. Conclusions: These findings support previous hypothesis that neural degeneration in ALS is not confined to the sole motor regions. In addition, the amount of cortical thinning in the temporal lobe helps to distinguish ALS patients from healthy controls, that is, to support or discourage the diagnosis of ALS, while the cortical thickness of the precentral gyrus specifically helps to distinguish the UMN from the LMN phenotype.

## 1. Introduction

Amyotrophic lateral sclerosis (ALS) is the fourth most common neurodegenerative disorder worldwide, with an incidence of approximately 3/100,000 person-years [1]. Clinically, ALS is characterized by the progressive paralysis of the motoneuron-innervated musculature and in most cases, becomes lethal within approximately three years since diagnosis due to respiratory insufficiency following respiratory muscle paralysis. ALS is not considered a single disease with an altered appearance, but rather an umbrella term to describe a number of phenotypic variants. From an anatomical point of view, clinicians distinguish ALS according to either the neural or somatic regions affected. In the former case, the distinction is made on whether the upper or the lower motor neuron (UMN and LMN) is predominantly affected; in the latter case, the difference is between a bulbar and a limb form [2]. Phenotypic variants of ALS may also occur as a multisystem degeneration, for example in patients additionally displaying symptoms of frontotemporal dementia (FTD-ALS) [3,4]. The degeneration of the UMN (or other brain regions) can only be assessed clinically [2,5,6], and specific biomarkers of UMN degeneration are still missing [7]. Brain and spine imaging is indeed recommended in the workup of ALS patients, but predominantly, to exclude other pathologies, such as myelopathies and spinal and/or brainstem tumours [8].

In a scientific context, different imaging modalities and statistical approaches, such as cortical thickness analysis (CT-A) [9,10,11], voxel-based morphometry and diffusion tensor imaging [7,12,13,14], have been applied to detect UMN pathology. So far, CT-A studies have revealed specific patterns of gray matter atrophy, for example in the precentral gyrus (PCG). ALS patients with the predominant affection of the UMN displayed a more pronounced cortical thinning in PCG than LMN patients and healthy controls (HeaCON), while no significant differences were found between the LMN phenotype and HeaCON. Patients with classical ALS fell somewhere between UMN and LMN ALS, showing a significant thinning of the PCG but not as pronounced as in the UMN phenotype [9,10,15]. Further, in patients with UMN-ALS, cortical thinning in the PCG seemed to develop early in the disease course; therefore, this parameter could become a precocious biomarker to predict a later clinical diagnosis of UMN degeneration. Cortical thinning in brain regions outside the motor system has also been described in ALS with UMN and LMN phenotypes. The most common finding is a decrease in cortical thickness (CT) in the temporal lobes (the left parahippocampal region and fusiform cortex [1], bilateral inferior temporal region and right middle temporal region [16], superior and inferior temporal regions [17], superior temporal gyrus [18] and left temporal gyrus [19]). Additionally, the reduction of CT has been described in various brain regions, such as the occipital lobes [16,17,19], the frontal [18] and cingulate [20] cortices, as well as parietal regions.

Current statistical brain imaging methods play only a minor role in the clinical workup of ALS patients, although the detection of specific patterns of brain atrophy could potentially contribute to the diagnosis of ALS. In the first step, structural magnetic resonance imaging (MRI) and CT-A based on high-resolution T1-weighted images to detect specific patterns of brain atrophy, that is, commonalities and differences in ALS patients with UMN and LMN phenotypes, are applied. However, univariate statistical analyses are not suited to provide subject-specific information. Against this background, more sophisticated statistical methods capable of supporting the diagnostic workup for individual patients are needed. To this end, we applied, in second step discrimination, analyses to investigate whether specific patterns of cortical thinning in and outside the motor system can be identified, enabling the distinctions as following: (i) between ALS patients and HeaCON and, within the ALS group; (ii) between UMN and LMN patients. A high classification accuracy would encourage the implementation of MRI and multivariate pattern analysis in the workup of ALS, especially since the performance of brain imaging is recommended anyway prior to the diagnosis of ALS, currently with the objective of the exclusion of other pathologies.

## 2. Methods

### 2.1. Patients and Controls

All patients were prospectively recruited from the Department of Neurology of the Bergmannsheil Clinic (Ruhr University Bochum, Bochum, Germany). The inclusion criteria were as follows: definite, probable, or possible diagnosis of ALS as defined by the Airlie House criteria [21], predominant affection of either the UMN or the LMN and absence of clinically relevant evidence for cognitive and behavioural impairment suggesting presence of a frontotemporal dementia. Evidence for UMN affection was clinically provided by the presence of pseudobulbar symptoms, hyperreflexia and/or spasticity and in the absence of muscular atrophy and hyporeflexia. The absence of UMN findings, together with clinical and/or neurophysiological symptoms of LMN affection, resulted in the diagnosis of LMN ALS.

The following exclusion criteria were applied: the presence of long-time arterial hypertension, cerebrovascular diseases, inflammatory diseases of the central nervous system, contraindications for MRI or inability to tolerate MRI testing. Of the 48 ALS patients initially examined, 10 were not included in the study because of movement artefacts in the MRI or the presence of cerebrovascular lesions (e.g., subcortical arteriosclerosis). All ALS patients were assessed using the revised Amyotrophic Lateral Sclerosis Functional Rating Scale (ALSFRS-R) [22] to quantify the severity of functional impairment. Following the Airlie House criteria, 6 patients had definite, 15 had probable, and 17 had possible ALS. Twenty-six HeaCON from a pre-existing database of the Bergmannsheil Clinic in Bochum served as the control group (WELDOX II study [23]).

All participants provided written informed consent for study inclusion prior to enrolment. The study was approved by the ethics committee of Ruhr University Bochum, Germany on 8 February 2013 (registration NO. 4413-12) and was conducted in accordance with the Declaration of Helsinki.

### 2.2. Data Acquisition

MRI was performed using a 3.0 Tesla scanner (Philips Achiva 3.2, Best, The Netherlands, with a 32-channel head coil). From all subjects, a T1-weighted data set were acquired: repetition time (TR), 8.3 ms; echo time (TE), 2.9 ms; flip angles, 8° and 9°, respectively; the field of view (FOV), 176 × 240 × 176 m^3^ (RL/AP/FH) for patients and 188 × 240 × 220 m^3^ for HeaCON, yielding 176 transversal slices with a voxel size of 1.0 × 1.0 × 1.0 m^3^. Images were transferred from the scanner to a Windows workstation and converted from DICOM to NIfTI format using “MRIconvert 2.0” (Lewis Centre for Neuroimaging, University of Oregon, Eugene, OR, USA).

### 2.3. Data Preprocessing

CT-A was performed automatically using the software Freesurfer (version 5.3, http://surfer.nmr.mgh.harvard.edu/, accessed on 24 October 2016), a tool freely available for download and described in detail under https://surfer.nmr.mgh.harvard.edu/fswiki/FreeSurferMethodsCitation, accessed on 24 October 2016. The processing stream generated thickness measures from 68 cortical and subcortical regions of interest (ROI), that is, 34 per hemisphere.

### 2.4. Statistical Analysis

As the grey matter volume and the CT in homologous brain regions tend to be highly correlated between the two hemispheres and the literature indicates that the left hemisphere is usually more affected by the neurodegenerative process compared to the right one [14], the focus remained on the left hemisphere. In the first step, comparisons between the three study groups (UMN, LMN and HeaCON) were performed. For each ROI, we determined whether the values were normally distributed. As this was not the case in some regions (for details, see Table 1), medians were reported (instead of means), and non-parametric tests were performed to determine group differences.

As in this study LMN patients tended to be older than UMN subjects and HeaCON (see results), a Quade test (equivalent to a non-parametric ANCOVA) with age as a covariate of no interest was performed. Bonferroni correction for multiple comparisons was performed for the number of ROIs (total number of ROIs: 34; 12 ROIs within the frontal lobe, 5 ROIs within the limbic lobe, 4 ROIs within the occipital lobe, 5 ROIs within the parietal lobe and 8 ROIs within the temporal lobe).

In the second step, we predicted group membership by performing discriminant analyses based on ROIs’ CT (independent variable), aiming to assess whether T1-weighted images and CT could be used to support or discourage the diagnosis of ALS. For each classification, a leave-one-out cross-validation (as a means to validate the reliability of the classifier) was performed; each case was deleted in turn from the training sample and classified by means of the classification rules established on the remaining observations. To this end, discriminant analyses were performed to distinguish the following: (1) the ALS group (including UMN and LMN patients) from the HeaCON group (classifications A1 and B1) and (2) between UMN, LMN and HeaCON (classifications A2 and B2). The following two approaches were used.

Analyses A1 and B1: According to the literature, the following three regions were entered to show cortical thinning in ALS patients: the left precentral gyrus, the left inferior temporal gyrus and the left temporal pole [9,10,15,16,17,19].

Analyses A2 and B2: A stepwise selection from all 34 regions of the left hemisphere was performed. Starting with an empty model, each step involved the inclusion of the ROI that yielded the maximum increase in the model probability, until a specific entry criterion was exceeded (F > 3.84, as suggested by SPSS).

## 3. Results

### 3.1. Subjects

The mean age and the male/female ratio of our patient population corresponded to those reported by epidemiologic studies on ALS. ALSFRS-R-scores were similar in the UMN and LMN groups, indicating equivalent clinical impairment in the two ALS cohorts. No differences in the male/female ratio (x^2^(1) = 2.186; *p* = 0.139) and ALSFRS-R-scores (x^2^(1) = 1.628; *p* = 0.202) were found between the UMN and LMN groups. The HeaCON group consisted of men only (see the limitation section). Age did not differ significantly between UMN, LMN and HeaCON (x^2^(2) = 5.672; *p* = 0.059). However, as there was a tendency for LMN subjects (mean age: 65 years; SD: 12 years) to be older than UMN patients (mean age: 57.5 years; SD: 18 years) and HeaCON (mean age: 56.5 years; SD: 14.8 years), age has been added as a covariate of no interest to the statistical models. For details on demographic and clinical data, see Table 2.

### 3.2. CT

**Figure 1:**
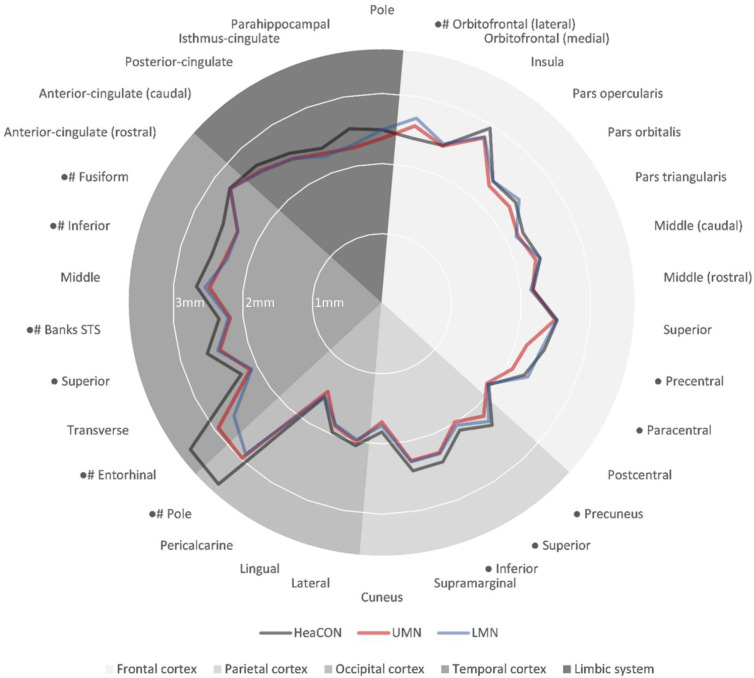
Cortical thickness of 34 brain regions from the left hemisphere for healthy controls (HeaCON indicated by the grey line), upper motor neuron (UMN indicated by the red line) and lower motor neuron patients (LMN indicated by the blue line). The grey shades depict different lobes, i.e., the frontal lobe, parietal lobe, occipital lobe, temporal lobe and limbic lobe. Significant differences between UMN patients and HeaCON are marked with a “●”, and those between LMN patients and HeaCON are marked with a “#”. HeaCON, healthy controls; LMN, lower motor neuron patients; UMN, upper motor neuron patients.

The univariate Quade test revealed differences in CT within 12 ROIs in the left frontal, parietal and temporal cortices (see Figure 1).

**Figure 2:**
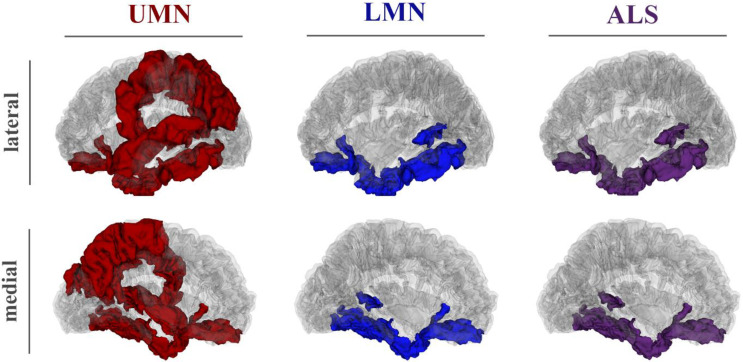
Differences in cortical thickness. Brain regions of the left hemisphere showed differences in cortical thickness compared to healthy controls identified using the Quade test plotted on a transparent MNI-template. Differences are shown either in red (UMN vs. HeaCON), in blue (LMN vs. HeaCON) or in violet (UMN and LMN vs. HeaCON). MNI, Montreal Neurological Institute; HeaCON, healthy controls; LMN, lower motor neuron patients; UMN, upper motor neuron patients.

Compared to the HeaCON, the ALS group (including UMN and LMN patients) showed reduced CT in the temporal pole, entorhinal cortex, superior temporal sulcus, inferior temporal gyrus and fusiform gyrus. Increased CT was observed within the lateral orbitofrontal cortex in patients with UMN and LMN (Figure 2).

In contrast, the reduced thicknesses of the precentral and paracentral gyrus, precuneus, superior parietal lobule and superior temporal gyrus were observed only in patients with UMN compared to in patients with LMN and HeaCON. Furthermore, a CT reduction for UMN compared to for HeaCON was found within the inferior parietal lobule, without a significant difference between the two ALS groups (Figure 2, left). For further information regarding CT and statistical results, see Table 3.

### 3.3. Classification—Discriminant Analyses

In total, 4 discriminant analyses were performed:

A1: ALS_Total_ vs. HeaCON (based on the CTs of the left PCG, inferior temporal gyrus and temporal pole);

A2: UMN vs. LMN vs. HeaCON (based on the CTs of the left PCG, inferior temporal gyrus and temporal pole);

B1: ALS_Total_ vs. HeaCON (stepwise CTs of 10 regions in the final model);

B2: UMN vs. LMN vs. HeaCON (stepwise, the CTs of five regions in the final model).

Discriminant analyses A1 (ALS vs. HeaCON) based on the left precentral gyrus (r = 0.114), the left inferior temporal gyrus (r = 0.553) and the left temporal pole (r = 0.811) revealed that 95% of the individuals could be classified correctly (see Table 4).

**Figure 3:**
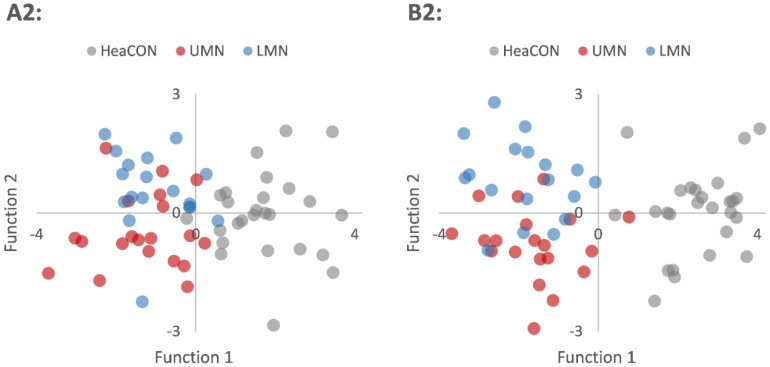
Functions of discriminant analyses. Discriminant functions for distinguishing between UMN, LMN and HeaCON based on the cortical thicknesses of either the precentral gyrus, inferior temporal gyrus and temporal pole (**A2**) or the precentral gyrus, temporal pole, lateral orbitofrontal gyrus, lingual gyrus and entorhinal cortex (**B2**-stepwise). HeaCON, healthy controls; LMN, lower motor neuron patients; UMN, upper motor neuron patients.

Discriminant analyses A2 (UMN vs. LMN vs. HeaCON) reached a cross-validated accuracy of 81%. Here, the correlations between the outcomes and the discriminant functions revealed that the CTs of the inferior temporal gyrus (r_1_ = 0.535, r_2_ = −0.357) and the temporal pole (r_1_ = 0.801, r_2_ = −0.239) were higher on the first function (r_1_), while the CT of the precentral gyrus (r_1_ = 0.167, r_2_ = 1.029) was higher on the second function (r_2_). The discriminant function plot showed that the first function discriminated both ALS groups from HeaCON, while the second one distinguished UMN from LMN (see Figure 3A2).

Discriminant analyses B1 (stepwise: ALS vs. HeaCON) reached a cross-validated classification accuracy of 100%. Ten regions contributed to the final model: the temporal pole (r = 0.439), lateral orbitofrontal cortex (r = −1.15), postcentral gyrus (r = −0.525), inferior parietal lobule (r = 0.728), lingual gyrus (r = 0.831), temporal pole (r = 0.863), entorhinal cortex (r = 0.411), transverse temporal gyrus (r = −0.374), inferior temporal gyrus (r = 0.449) and anterior cingulate cortex (r = −0.572). Discriminant analyses B2 (stepwise: UMN vs. LMN vs. HeaCON) achieved a classification accuracy of 82.8%. Five regions contributed to the final model: the lateral orbitofrontal cortex (r_1_ = −0.988, r_2_ = −0.036), precentral (r_1_ = 0.153, r_2_ = 1.12), lingual gyrus (r_1_ = 0.682, r_2_ = −0.452), temporal pole (r_1_ = 0.818, r_2_ = 0.182) and entorhinal cortex (r_1_ = 0.342, r_2_ = −0.684). While the first function (r_1_) discriminated between patients with ALS and healthy controls (see Figure 3B2) and relied strongly on the lateral orbitofrontal cortex, lingual gyrus and temporal pole, the second function (r_2_) relied on the precentral gyrus and the entorhinal cortex, thereby separating the UMN from LMN patients (see Figure 3B2). For further details, see Table 4 and Table 5.

## 4. Discussion

In this study, high-resolution structural MRI and CT-A were utilized to compare ALS patients with either UMN or LMN manifestation to a group of HeaCON. As the main finding, both ALS groups displayed significant cortical thinning in the temporal lobe, more specifically in the temporal pole, entorhinal cortex, fusiform gyrus, inferior temporal gyrus and banks of the superior temporal sulcus. In addition to these findings, the UMN group also showed cortical thinning in the PCG, prefrontal, superior frontal and ventral frontal cortices, superior and inferior parietal regions, medial and lateral occipital areas, cingulate gyrus and insula. Significant differences between the UMN and LMN groups, in terms of the reduced CT in the UMN patients, were observed in the PCG, paracentral lobule and precuneus. Interestingly, both ALS groups displayed increased CT in the orbitofrontal cortex. By performing multivariate analyses, a high accuracy was achieved when distinguishing between ALS patients and HeaCON (>95%), and a moderate accuracy was obtained when distinguishing between all three groups, that is, UMN, LMN and HeaCON (>80%).

The results of this study are in line with other studies using CT-A in ALS, which have already reported a pronounced thinning of the PCG in patients with UMN disease, in contrast to a non-significant cortical thinning in ALS patients with the LMN phenotype and in ALS mimic disorders [9,10,15]. In both UMN and LMN patients, we found cortical thinning in the temporal lobe, while the UMN patients displayed additional regions with CT reduction (i.e., the medial and lateral parietal lobes). Likewise, Grieve et al. found significant CT reductions in the inferior temporal region bilaterally and in the right middle temporal gyrus of patients with ALS [16], but no cortical thinning in the PCG. Importantly, the study by Grieve et al. did not distinguish between UMN and LMN phenotypes. Possibly, subjects with a predominant LMN phenotype might have diluted down cortical thinning in the PCG of this mixed cohort. Interestingly, Walhout et al. found that asymptomatic carriers of the C9orf72-repeat expansion (a genotype of familial ALS) show cortical thinning in the temporal and parietal lobes, but not in the PCG, when compared to non-carriers of the same family [24]. Interestingly, within a longitudinal study, Walhout et al. described the cortical thinning of the temporal lobes, applying CT-A to a mixed ALS population consisting of patients with classical ALS, UMN phenotype and LMN phenotype, as compared to ALS mimic disorders [15]. Likewise, Schuster et al. reported that dominant UMN patients demonstrate the most distinct thinning of the PCG, followed by classical ALS patients, and that pure LMN variants do not differ from HeaCON [9,25].

As such, the present study suggests, in agreement with the previous literature, involvement of the temporal lobe in ALS with both UMN and LMN phenotypes, supporting the hypothesis that the degenerative process of ALS also involves non-motor brain regions and is not confined to only the motor system. To confirm this, similarities in cortical thinning (mainly frontally) between FTD, FTD-ALS and ALS have been recently detected, suggesting a continuum in brain morphology from pure motor ALS to FTD [3].

In summary, the data suggest that both UMN- and LMN-ALS display changes in brain morphology in terms of a cortical thinning in the temporal lobe and, in the case of the UMN patients, additionally in the parietal lobe and the PCG. Neuropathological correlation is still speculative. The loss of Betz cells [11] and inhibitory cortical interneurons [26] observed in ALS do not explain the morphological changes outside the motor cortex. Neuroimaging (MRI and PET) has shown a diffuse hypo metabolism in the frontal and anterior cingulate cortex [26], and in this case, the underlying mechanisms of the cortical thinning in the temporal lobe remain unclear. From a diagnostic point of view, it will be interesting to evaluate whether these changes are suited to serve as diagnostic criteria. The diagnosis of ALS requires signs of UMN involvement. The PCG is directly related to UMN, and as such, it is plausible that PCG atrophy supports the clinical diagnosis of UMN-ALS. However, the interesting question is whether the CT of the PCG might serve as an imaging marker of the UMN pathology in the absence of clinical UMN signs. This is unlikely, as in that case patients would be classified as LMN patients; specifically, for that patient group, no CT alterations in the PCG were found. As such, the CT-A of the PCG is not suited to unravel UMN pathology in (true) LMN patients, possibly because the UMN is not affected. On the other hand, given that cortical atrophy in regions outside the motor system, that is, in the temporal lobe, is accepted to be typical or representative of ALS, CT-A holds promise as a valuable add-on to the clinical workup of ALS. As such, it is conceivable that the detection of temporal lobe atrophy is indicative of CNS involvement, supporting the diagnosis of ALS. To this end, we performed multivariate pattern analysis, that is, discrimination analysis, based on the regional CTs of 34 brain regions (as suggested by the Freesurfer algorithm) to distinguish ALS patients from HeaCON. In the first step, three regions known to show cortical thinning in ALS were selected (i.e., the PCG, inferior temporal gyrus and temporal pole), reaching an accuracy of approximately 95% when distinguishing ALS patients from the HeaCON and an accuracy of about 80% when distinguishing among all the three groups. Interestingly, when applying the stepwise method, where the classifiers picked the regions that contribute the most to high accuracy, the PCG did not play a significant role in the distinction between ALS (UMN + LMN) and HeaCON. At least 10 other regions were found to be more robust. However, when it came to distinguishing among all three groups, the PCG was among the five most robust regions, specifically contributing, such as the entorhinal cortex, to the distinction between UMN and LMN patients.

Limitations: This study has some limitations that need to be addressed. First, the differences were investigated in regional brain morphology between UMN and LMN-ALS. The distinction between these two phenotypes and classical ALS is somewhat heterogeneous. While some authors make a diagnosis of UMN-ALS only in the strict absence of any signs of LMN degeneration (including EMG findings) [16], others allow the coexistence of mild signs of LMN (e.g., fasciculation), given that UMN involvement is clearly predominant. As such, this classification was based on clinical phenotype predominance, and some of the UMN patients might have been classified as “classical ALS” in other studies. Second, the present study was cross-sectional. As such, group differences were reported at a given time point; this study design does not allow for the assessment of dynamic changes, and longitudinal studies are required to determine the rate of cortical thinning in different brain regions at different stages of the disease. Third, the two ALS groups and the control group differed in their age and sex ratios. The patients with LMN were older than those with UMN or HeaCON. For univariate group comparisons, age was added as a nuisance variable, such that these findings are unlikely to be age-related. For the multivariate pattern analyses, it will be interesting to evaluate if the comparisons to age-adjusted groups of HeaCON (e.g., comparing an individual patient to a group of HeaCON in an age range of ±5 years) yields similar accuracies. The control group consisted of men only; as the scanner underwent an upgrade just after the recruitment of ALS patients had been finished and scanner upgrades tended to have an impact on acquisition parameters, we decided to rely on a control group that had already been acquired in the months before. It must be noted, that gender differences have been described in the preclinical ALS model, but also in human cohorts, specifically considering clinical development (females being more protected against ALS than males), disease severity and progression, but also potential pathogenic mechanisms underlying ALS [27,28]. To our knowledge, sex-related differences in CT have only been described, so far, in a small population of 14 female and 13 male ALS patients. While female ALS showed a trend towards higher age-adjusted CTs in the right parieto-occipital and left mid-frontal regions compared to healthy females and males with ALS, males with ALS demonstrated higher CTs in the left lingual and left superior temporal regions compared to healthy males and females with ALS [29]. Because of the small cohort, the results should be interpreted with caution. Nevertheless, the study suggests the importance of a gender-related study design when investigating structural brain changes in ALS. Fourth, the cognitive status of our patients and HeaCON has not been specifically investigated. Cognitive impairment in ALS is an issue of increasing interest. In this study, none of the patients displayed evidence of severe cognitive or behavioural symptoms. However, a proper neuropsychological assessment has not been performed. Against the background that the temporal lobe plays an important role in various cognitive functions and that ALS is considered to be clinically related to frontotemporal dementia [2,30], future studies should investigate in more detail how patterns of brain atrophy relate to cognitive impairment and whether this might add additional accuracy to MRI-supported diagnoses of ALS.

Overall, the findings of the current study need to be validated within larger groups. Even more importantly, for the assessment of classification accuracy, the application of the classifier to a completely independent group is vital. As such, the leave-one-out method can only create a first impression. Nevertheless, with an accuracy of >95%, our approach can at least be regarded as promising.

## 5. Conclusions

The results of this study provide strong evidence for the cerebral involvement of brain regions outside the motor system in ALS, that is, in the temporal lobe in both UMN and LMN phenotypes as a common feature, and additionally in the PCG and the parietal lobe in UMN patients. Furthermore, based on CT-A, the correct distinction of ALS patients from the HeaCON could be performed with an accuracy of approximately 95% using a leave-one-out approach. Applying a rather simple algorithm, that is, discrimination analysis, the current study was meant to contribute to the implementation of quantitative brain imaging to the radiological workup of ALS patients, providing specific parameters that can be used to either encourage or discourage the diagnosis of ALS and even suggest specific subtypes. Given that an accuracy of >90% has been reached in larger and independent samples, this approach holds promise as a useful and valid tool in the workup of ALS patients.

## Figures and Tables

**Table 1:** Normality distribution of cortical thickness within regions of interest. Results from the Shapiro–Wilk test for the normality distribution for each ROI in the left hemisphere per group. Values below 0.05 indicate non-normally distributed data. HeaCON, healthy controls; LMN, lower motor neuron patients; UMN, upper motor neuron patients.

Localization		Distribution		
Cortex/Areal		HeaCON	UMN	LMN
Frontal	Pole	0.481	0.404	0.211
	Orbitofrontal-lateral	0.016	0.820	0.241
	Orbitofrontal-medial	0.329	0.224	0.025
	Insula	0.991	0.291	0.683
	Pars opercularis	0.523	0.794	0.732
	Pars orbitalis	0.502	0.920	0.562
	Pars triangularis	0.521	0.036	0.623
	Middle-caudal	0.955	0.902	0.979
	Middle-rostral	0.837	0.195	0.160
	Superior	0.730	0.246	0.261
	Precentral	0.905	0.451	0.037
	Paracentral	0.472	0.954	0.364
Parietal	Postcentral	0.219	0.206	0.129
	Precuneus	0.413	0.029	0.431
	Superior	0.427	0.703	0.061
	Inferior	0.452	0.971	0.222
	Supramarginal	0.566	0.287	0.043
Occipital	Cuneus	0.464	0.705	0.293
	Lateral	0.561	0.417	0.552
	Lingual	0.261	0.786	0.897
	Pericalcarine	0.434	0.745	0.375
Temporal	Pole	0.043	0.009	0.446
	Entorhinal	0.344	0.921	0.901
	Transverse	0.935	0.796	0.335
	Superior	0.660	0.095	0.290
	Banks of superior sulcus	0.888	0.798	0.739
	Middle	0.173	0.864	0.123
	Inferior	0.637	0.443	0.575
	Fusiform	0.959	0.350	0.531
Limbic	Anterior cingulate-rostral	0.870	0.611	0.994
	Anterior cingulate-caudal	0.322	0.670	0.319
	Posterior-cingulate	0.882	0.507	0.291
	Isthmus of cingulate	0.247	0.981	0.368
	Parahippocampal	0.041	0.173	0.760

**Table 2:** Demographic and clinical data of the three study groups including results from statistical x^2^ test.

		HeaCON	UMN	LMN	*p*-Value
Sex (male/female)	Sum:	26/0	11/9	14/4	0.139 *
Age (years)	Median:	56.5	57.5	65.5	0.059
	IQR:	14.8	18	13	
ALSFRS-R-score	Median:	-	35	39.5	0.202 *
	IQR:	-	7.5	8	

HeaCON, healthy controls; LMN, lower motor neuron; UMN, upper motor neuron; ALSFRS-R, revised Amyotrophic Lateral Sclerosis Functional Rating Scale. * involving only the UMN and LMN groups, - not applicable

**Table 3:** Statistical results (descriptive, univariate) of cortical thickness from 34 different cortical regions of the left hemisphere in HeaCON, LMN and UMN. Significances were Bonferroni-corrected for multiple comparisons. HeaCON, healthy controls; LMN, lower motor neuron patients; UMN, upper motor neuron patients.

Localization		Cortical Thickness (mm)						Statistics			
Cortex/Areal		HeaCON		UMN		LMN		Quade Test	Post-Hoc (*p*-Value)		
		Median	IQR	Median	IQR	Median	IQR	*p*-value	HeaCON–UMN	HeaCON–LMN	UMN–LMN
Frontal	Pole	2.48	0.3	2.36	0.2	2.48	0.2	1	-		
	Orbitofrontal-lateral	2.40	0.5	2.58	0.4	2.69	0.3	0.001	0.002	<0.001	0.936
	Orbitofrontal-medial	2.42	0.2	2.42	0.2	2.44	0.2	1	-		
	Insula	2.95	0.2	2.78	0.2	2.80	0.2	0.934	-		
	Pars opercularis	2.37	0.5	2.28	0.4	2.37	0.7	0.73	-		
	Pars orbitalis	2.40	0.2	2.29	0.2	2.47	0.2	1	-		
	Pars triangularis	2.26	0.2	2.18	0.2	2.15	0.2	1	-		
	Middle-caudal	2.36	0.2	2.29	0.2	2.36	0.2	1	-		
	Middle-rostral	2.18	0.3	2.17	0.3	2.14	0.3	1	-		
	Superior	2.53	0.2	2.50	0.2	2.52	0.2	1	-		
	Precentral	2.42	0.2	2.16	0.3	2.38	0.3	<0.001	<0.001	0.866	<0.001
	Paracentral	2.29	0.2	2.09	0.1	2.34	0.2	0.006	0.001	1	<0.001
Parietal	Postcentral	1.92	0.1	1.89	0.2	1.90	0.1	1	-		
	Precuneus	2.35	0.2	2.17	0.2	2.28	0.1	0.003	<0.001	0.793	0.007
	Superior	2.12	0.3	2.00	0.5	2.04	0.4	0.01	<0.001	0.538	0.032
	Inferior	2.42	0.2	2.28	0.2	2.29	0.2	0.01	<0.001	0.334	0.057
	Supramarginal	2.42	0.2	2.28	0.2	2.29	0.1	0.052	-		
Occipital	Cuneus	1.83	0.3	1.69	0.3	1.75	0.3	0.183	-		
	Lateral	2.06	0.2	2.00	0.2	1.97	0.2	1	-		
	Lingual	1.97	0.2	1.87	0.2	1.85	0.2	0.167	-		
	Pericalcarine	1.57	0.2	1.48	0.1	1.55	0.1	1	-		
Temporal	Pole	3.48	0.2	3.00	0.2	2.90	0.1	<0.001	<0.001	<0.001	1
	Entorhinal	3.45	0.2	2.94	0.2	2.66	0.1	<0.001	<0.001	<0.001	0.92
	Transverse	2.26	0.2	2.11	0.2	2.10	0.2	1	-		
	Superior	2.61	0.3	2.42	0.4	2.45	0.2	0.012	<0.001	0.107	0.207
	Banks of superior sulcus	2.35	0.1	2.18	0.2	2.21	0.2	0.023	0.001	0.014	1
	Middle	2.67	0.2	2.48	0.2	2.55	0.2	0.059	-		
	Inferior	2.54	0.2	2.34	0.2	2.31	0.2	<0.001	<0.001	<0.001	1
	Fusiform	2.54	0.3	2.31	0.2	2.31	0.2	<0.001	<0.001	<0.001	0.765
Limbic	Anterior-cingulate-rostral	2.73	0.2	2.73	0.3	2.73	0.2	1	-		
	Anterior-cingulate-caudal	2.67	0.3	2.57	0.4	2.55	0.2	1	-		
	Posterior-cingulate	2.52	0.6	2.43	0.3	2.43	0.4	1	-		
	Isthmus of cingulate	2.38	0.4	2.30	0.3	2.29	0.3	1	-		
	Parahippocampal	2.54	0.2	2.26	0.3	2.30	0.2	1	-		

**Table 4:** Discriminant analyses. Predicted group membership and leave-one-out cross-validation for discriminant analyses involving either the left precentral gyrus, inferior temporal gyrus, and temporal pole (A) or 34 ROIs from the left hemisphere using stepwise selection (B). Discriminant analyses were performed to distinguish between ALS (UMN and LMN) and HeaCON (1) or between UMN, LMN and HeaCON (2). HeaCON, healthy controls; LMN, lower motor neuron patients; UMN, upper motor neuron patients.

Group		Prediction			Validation
		HeaCON	UMN	LMN	Leave-One-Out
A1	HeaCON	96.2%	3.8%		95.3%
	ALS	2.6%	97.4%		
A2	HeaCON	96.2%	0%	3.8%	81.2%
	UMN	0%	70%	30%	
	LMN	5.6%	11.1%	83.3%	
B1	HeaCON	100%	0%		100%
	ALS	0%	100%		
B2	HeaCON	100%	0%	0%	82.8%
	UMN	5%	80%	15%	
	LMN	0%	22.2%	77.8%	

**Table 5:** Functions of discriminant analyses. Standardized canonical function coefficients (r) of the discriminant analyses for all involved ROIs. If the discriminant analysis provides a distinction between UMN, LMN and HeaCON (2), the coefficients for both functions are shown (r_1_, r_2_). For stepwise analyses (B) the step number in which the ROI was added is given (n-step). HeaCON, healthy controls; LMN, lower motor neuron patients; UMN, upper motor neuron patients.

Localization		Discriminant Analyses							
Cortex/Areal		A1	A2		B1		B2		
		r	r_1_	r_2_	n-Step	r	n-Step	r_1_	r_2_
Frontal	Pole	-	-		6	0.439	-		
	Orbitofrontal-lateral	-	-		2	−1.15	2	−0.988	−0.036
	Precentral	0.114	0.167	1.03	-		4	0.153	1.12
Parietal	Postcentral	-	-		10	−0.525	-		
	Inferior	-	-		9	0.728	-		
Occipital	Lingual	-	-		3	0.831	3	0.682	−0.452
Temporal	Pole	0.811	0.801	−0.239	1	0.863	1	0.818	0.182
	Entorhinal	-	-		4	0.411	5	0.342	−0.684
	Transverse	-	-		8	−0.374	-		
	Inferior	0.553	0.535	−0.357	7	0.449	-		
Limbic	Anterior-cingulate-rostral	-	-		5	−0.572	-		

## Data Availability

The data that support the findings of this study are available from the corresponding author upon reasonable request.

## Abbreviations

ALS	amyotrophic lateral sclerosis
ALSFRS-R	revised Amyotrophic Lateral Sclerosis Functional Rating Scale
CT	cortical thickness
CT-A	cortical thickness analysis
FOV	field of view
FTD	frontotemporal dementia
HeaCON	healthy controls
LE	lower extremity
LMN	lower motor neuron
MRI	magnetic resonance imaging
PCG	precentral gyrus
ROI	region of interest
UE	upper extremity
UMN	upper motor neuron
